# Assessing the Effectiveness of Tobacco 21 Laws to Reduce Underage Access to Tobacco: Protocol for a Repeated Multi-Site Study

**DOI:** 10.3390/mps6020027

**Published:** 2023-03-10

**Authors:** Mary Hrywna, Amanda Y. Kong, Christopher Ackerman, Daniel P. Giovenco, Torra E. Spillane, Joseph G. L. Lee, Shawna V. Hudson, Cristine D. Delnevo

**Affiliations:** 1 Rutgers Center for Tobacco Studies, Rutgers University, New Brunswick, NJ 08901, USA; 2 Department of Health Behavior, Society & Policy, Rutgers School of Public Health, Piscataway, NJ 08854, USA; 3 Department of Family and Preventive Medicine, University of Oklahoma Health Sciences Center, Oklahoma City, OK 73104, USA; 4 TSET Health Promotion Research Center, Stephenson Cancer Center, University of Oklahoma Health Sciences Center, Oklahoma City, OK 73104, USA; 5 Department of Sociomedical Sciences, Columbia University Mailman School of Public Health, New York, NY 10027, USA; 6 Department of Health Education and Promotion, College of Health and Human Performance, East Carolina University, Greenville, NC 27858, USA; 7 Department of Family Medicine & Community Health, Rutgers Robert Wood Johnson Medical School, Rutgers, The State University of New Jersey, New Brunswick, NJ 08901, USA

**Keywords:** tobacco, e-cigarettes, cigarettes, cigars, smokeless tobacco, tobacco-free nicotine pouches, youth, young adults, policy, prevention

## Abstract

Prior to the federal law passed in December 2019, many states passed an increased age of sale law prohibiting youth under age 21 (or Tobacco 21) from purchasing tobacco products, including e-cigarettes. Although previous research has documented tobacco retail sale violations, fewer studies have examined age verification and illegal tobacco sales in the context of Tobacco 21 or repeated purchase attempts in various settings. In this study conducted between 2019 and 2022, buyers aged 18 to 20 years made repeated unsupervised purchase attempts of cigarettes, cigars, e-cigarettes, tobacco-free nicotine pouches, and smokeless tobacco at over 180 tobacco or e-cigarette retailers in New Jersey, New York City, and Pitt County (North Carolina). Buyers documented whether they were asked for identification and whether they were able to successfully purchase a tobacco or nicotine product at each visit. The primary outcome will be the percent of retailers that checked the buyers’ identification at store visits and the percent of visits that resulted in a successful underage tobacco product purchase. We will compare the results across time periods, study sites, products, and buyer characteristics (i.e., gender, minoritized racial/ethnic identity) while controlling for repeated visits. These results will inform improvements to current compliance check inspection programs as well as interventions that reduce youth access to tobacco.

## 1. Introduction

Despite notable reductions in cigarette smoking in the United States (U.S.), concerns regarding tobacco use among youth and young adults remain [1]. Experimentation and initiation before the age of 18 have been a strong predictor of adult daily tobacco use; 87% of adults who had ever smoked cigarettes daily tried their first cigarette by the age of 18 [2]. As such, efforts to delay tobacco initiation and decrease experimentation are high priorities for tobacco control. In particular, increasing the minimum legal sales age for tobacco products to age 21, or Tobacco 21 (T21), has been adopted as a promising strategy to delay tobacco initiation and reduce tobacco use prevalence among youth.

A 2015 Institute of Medicine (IOM) report concluded that raising the minimum tobacco sales age to 21 nationwide would reduce smoking initiation over time by 25% among 15- to 17-year-olds and by 15% among 18- to 20-year-olds [3]. The reduction in tobacco use initiation achieved by T21 would be the result of disrupting the acquisition channel for underage users by reducing social access to tobacco via older peers and interrupting the trajectory from experimentation to regular use [3]. While about 30 localities (primarily in Massachusetts) adopted T21 before 2015, the IOM report accelerated diffusion, leading to over 500 local and 19 state laws before culminating in the federal T21 law being passed in December 2019 [3,4,5].

The extent to which T21 will reduce youth access depends on consistent implementation. An important question remains as to whether the T21 law improves age of sale compliance among retailers, both by increasing requests for proof of age and decreasing sales to underage buyers. Recent observational data from early adopters of T21 policies provides evidence that such policies may curb rates of youth and young adult tobacco use, including e-cigarettes [6,7,8,9,10,11], but the limited data available from retail compliance checks are mixed and suggest that policies are not uniformly enforced [12,13,14,15]. Following New York City’s T21 law, retailer compliance with identification (ID) checks of legal age buyers actually declined from 71.0% to 61.4%, with chain store retailers having higher rates of compliance versus independent stores [13]. After T21 became effective in California, youth (aged 15–16) were successful in 5.7% of buy attempts for traditional tobacco (i.e., cigarettes, little cigars or cigarillos, big cigars, or chewing tobacco), while 14.2% and 13.2% of young adults (aged 18–19) succeeded in buying traditional tobacco and e-cigarette products, respectively; in this California study, tobacco and vape shops were more likely to make an illegal sale [14]. In a study conducted among 91 retailers in Columbus, Ohio, in both 2017 and 2018, researchers found that 39% of retailers conducted ID checks before T21 compliance, and this increased to 78% in the year after the citywide implementation of the T21 law [15].

However, previous research suggests that single compliance checks may not provide accurate estimates of retailer compliance [16,17]. While underage buys have been widely used to measure cigarette sales to minors, there are numerous methodological limitations of current underage buyer protocols that ultimately limit the ecological validity of their findings [18]. A “familiarity protocol” is recommended to reflect real-world purchasing behaviors, including the use of buyers with tobacco use experience who look and act like tobacco users and repeated visits to the same store with the same buyer [19]. Repeated visits are thought to improve measurement as a single purchase is subject to variability based on the time of day, the clerk on duty, and other contextual factors [17].

We describe a protocol for a tobacco purchase study conducted across three sites (New Jersey [NJ], New York City [NYC], and Pitt County, North Carolina [NC]) to assess whether tobacco retailers asked for proof of age from and/or sold tobacco products to underage purchasers.

## 2. Design

We employed a fractional factorial design (3 to 5 separate tobacco product purchase attempts × 2 genders × 2 racial/ethnic identities) at each site. All sites attempted separate purchase attempts of cigarette, cigar/cigarillo, and e-cigarette products at each point in time. There were minor variations in the number of products by sites and over time: Smokeless tobacco was included solely in NC due to the low prevalence of use and product availability in NJ and NYC. Smokeless product purchases were only attempted by male buyers, given the extremely low prevalence of smokeless tobacco use among females [20,21,22] and the potential for these purchase attempts to unduly raise suspicion. Additionally, given the recent market growth of tobacco-free nicotine pouches, this product was added to all sites beginning in 2021.

### 2.1. Sample

This study began focusing solely on NJ, but given the expansion of federal T21, two locations: NYC and Pitt County, NC, were added over time. Table 1 summarizes sample sizes over time in all three locations. Details for each site’s sample are described below.

#### 2.1.1. New Jersey

The sampling frame in NJ included all licensed tobacco retailers in municipalities within a 25 mile radius of New Brunswick, New Jersey. In 2019, there were 4011 tobacco retailers within the selected area. We identified and removed 529 liquor stores from the sampling frame in NJ, given that regulations largely restrict retail alcohol sales to liquor stores only. We then randomly sampled 50 retailers from high population density municipalities (n = 1884) and 50 retailers from low population density municipalities (n = 1598). Prior to fielding, research staff completed store audits in person to confirm that stores were open and assessed the availability of all tobacco products, after which 14 stores were removed from the sample because of permanent closure (4), bar/liquor stores (4), safety concerns (3), or tobacco no longer for sale (3), leaving a final sample of 86 licensed tobacco retailers. In 2021, or Wave 2, we modified the sample to remove tobacco retailers that sold only one type of tobacco product, which resulted in an eligible sample of 65 stores. We also added vape shops within a 25-mile radius of New Brunswick, NJ. Availability audits were conducted, after which five stores were removed from the sample because of their permanent closure (2), no tobacco products sold (2), or only one tobacco product for sale (1), leaving a final sample of 60 stores and 10 vape shops. In 2022, or Wave 3, availability audits reduced the final sample of stores to 56 stores and 10 vape shops.

#### 2.1.2. North Carolina

To sample retailers in Pitt County, NC, which does not have state licensing of tobacco retailers, we used methods previously validated in NC [23]. We separately identified vape shops through Google Maps and Yelp, following an approach previously validated in Pitt County, NC [24]. To get to a sample of 50 retailers and up to 10 vape shops, a sample of 60 retailers was selected using a simple random sample in SPSS v28 Complex Samples, and this list was combined with a census of all vape shops (N = 12) in Pitt County. Of this list of 72, in-person audits were conducted, and 15 stores were removed from the sample because of permanent closures (7), duplicate records (4), being unable to locate (3), and safety concerns (1). This resulted in a sample of 57 retail locations, of which data collectors attempted purchases at 56. No attempts were made at one store, as it would have resulted in more than 50 retailers being included, and one vape shop ended up selling combustible products and was reclassified. This resulted in an analytic sample of 51 retailers that sold combustible products and a census of all five exclusive vape shops in Pitt County, NC. A second wave of visits involved 53 retailers due to store closures (49 retailers and four vape shops) and resulted in 663 purchase attempts.

#### 2.1.3. New York City

For NYC’s 2019 wave of data collection, or Wave 1, a simple random sample of 100 retailers was selected from a sampling frame of all licensed tobacco retailers in the borough of Manhattan (n = 1359). After conducting initial availability audits, a total of 92 retailers were operating and selling tobacco products and were included in the final sample (four stores were permanently closed and four could not be located). For NYC’s modified 2021 sample, or Wave 2, we used stratified random sampling to randomly select 10 retailers in 5 Manhattan neighborhoods that varied by socioeconomic and demographic characteristics for a total of 50 tobacco retailers. We also added six vape shops that were operating in Manhattan, for a total sample size of 56 retailers that were confirmed to be in operation and selling tobacco products. The modified sample for Wave 2 was selected to reduce travel time between stores (e.g., all neighborhoods accessible via subway), thereby improving efficiency and feasibility.

### 2.2. Covert Buyers

We recruited and trained covert buyers between the ages of 18 and 20. Given previous research suggesting that sales to minors varied by gender and race/ethnicity [18,25,26], attempts were made to recruit a racially, ethnically, and gender diverse team of buyers. Our intention was for each site to have a minimum of two males (non-Hispanic White and person of color) and two females (non-Hispanic White and person of color) who would visit all the stores, individually, to attempt purchases of each tobacco or nicotine product on separate occasions. Our buyer characteristics are detailed in Table 2. In some cases, buyers were retained over multiple waves of fielding if eligible at the time of fielding (i.e., between ages 18 and 20).

## 3. Procedure

The methods for data collection mirrored those from an earlier pilot study in New Jersey conducted around the time New Jersey’s T21 law became effective [27]. The Rutgers Health Sciences Institutional Review Board determined this study was non-human subject research. Data collectors from all sites completed a similar 2-day in-person training on study protocol, including safety measures. In separate visits, each covert buyer was assigned to attempt purchases of each product in every store where it was available. All sites made covert buy attempts for cigarettes, cigars, cigarillos, and e-cigarettes. Covert buys for smokeless tobacco only occurred in NC due to the low prevalence of use and product availability in NJ and NYC. Additionally, given the growth in tobacco-free nicotine pouches, this product was added to all sites starting in 2021. Purchases were attempted without any additional purchases (e.g., buyers were not asked to buy chips or a drink). We varied buyers’ schedules and product purchase attempts to eliminate overlap in store visits and reduce suspicion of compliance checks. Federal law requires carding anyone under the age of 27 who attempts to purchase tobacco. If a store clerk asked buyers about age, or more specifically, if they were 21, the buyers were instructed to say, “Do you want to see my ID?” Our buyers were instructed not to lie about age and to show their legal driver’s license if asked for ID. In addition to providing birthdate, most state driver’s licenses are printed on the vertical axis if the license holder is under 21, including in New Jersey and North Carolina. The buyers’ licenses in the NYC sample were from several states (i.e., California, Connecticut, Michigan, New Jersey, New Mexico, New York, Pennsylvania, and Virginia) that display vertically for minors. One buyer used their passport, an accepted form of identification for tobacco purchases in NYC. Buyers repeated purchase attempts in all stores, regardless of the outcomes of their prior attempts.

### 3.1. Measures

For each store visit, buyers recorded what tobacco product they attempted to purchase (i.e., cigarettes, cigars, e-cigarettes, tobacco-free nicotine pouches, or smokeless tobacco). Additionally, during our availability audit, each store type was classified (i.e., chain convenience, convenience [non-chain] bodega, drug store, supermarket/grocery store, gas station/kiosk, dollar store, vape/smoke shop, and other [e.g., fast food]). Stores classified as chain convenience were typically a franchised or corporately owned, had multiple locations with similar inventory and layout, and were often licensed under a parent company name like Quick Chek or WaWa, while non-chain convenience stores or bodegas were smaller, independently owned grocery/convenience stores with less diverse product inventory and were typically licensed under an individual name. Drug stores included all pharmacies, including both chain (e.g., Walgreens, Rite-Aid) and smaller independently owned pharmacies. Supermarkets or grocery stores included both chains and small, independently owned stores that primarily sold fresh and packaged foods and household items. Gas stations or kiosks were retailers primarily selling fuel but limited in other inventory (e.g., they did not also include convenience stores). Dollar stores were discount or value stores that sold a variety of inexpensive items, typically for a dollar or less, and often had the word “dollar” in the store or licensee name. Vape and smoke shops specialized in the retail sale of e-cigarette/vapor, and cigarette products; some shops focused primarily on one product type while other stores carried a range of combustible and non-combustible products.

After each store visit, buyers used an electronic survey to answer several questions, including whether they were able to attempt the purchase, the product they attempted to purchase, whether the store clerk asked for identification when attempting to purchase (including electronic verification), and whether they were successfully able to purchase. In addition, buyers noted the perceived age, race, and ethnicity of the store clerk. If they were able to purchase the product, they were also asked to provide the purchase price of the product. In 2021 and 2022, in the context of COVID-19, buyers were also asked to indicate if they wore a mask (i.e., yes/no) when attempting to purchase the product.

### 3.2. Data Analysis Overview

The study design will allow for analyses of between and within effects by time, geographic location, product type, and buyer characteristics. Our primary outcome variables are whether the store clerk asked to check a buyer’s identification (1 = yes, 0 = no) when attempting to purchase each tobacco product and whether buyers were successfully able to purchase each of the tobacco products (1 = yes, 0 = no). In addition, our initial analysis will test whether each explanatory variable was associated with either (1) an identification check or (2) a successful tobacco product purchase, regardless of whether identification was checked. In addition, we will fit models to test whether each explanatory variable (as described above, including product and store type) was associated with a successful purchase for those store visits where identification was checked. Because buyers attempted to purchase a single tobacco product type at each store visit, a single store could have been visited up to 15–20 times. Statistical model selection will depend on the specific purpose of each analysis, but planned analyses will account for repeated measurements or clustering as appropriate given the study design.

## 4. Expected Results

The primary study finding will be to estimate the extent to which purchase attempts resulted in the successful purchase of various tobacco products to assess compliance with the increased tobacco age of sale law to 21. Secondary findings will be whether other factors (e.g., store type, tobacco retailer density, electronic verification of ID, buyer sociodemographic characteristics) were associated with identification checks or successful purchases. We will aim to compare the results across time periods and study sites.

## 5. Discussion

To date, no studies have used a repeated purchase attempt protocol to objectively measure retailer compliance with Tobacco 21 laws across several sites and time periods. Studies investigating retailer compliance with age of sale laws have been limited to designs involving single purchase attempts, often in supervised visits, which do not reflect actual purchasing behavior [18]. In addition, these studies are often restricted to underage purchase attempts of cigarette products only [13,15,18]. Via our fractional factorial design, which leverages data from a diverse group of buyers in several sites in the eastern U.S., this study shows that it is feasible to collect data from repeated store visits to attempt a variety of tobacco product purchases across a variety of settings.

This study should be considered in the context of its limitations. First, the study may be underpowered to detect some associations; for example, in the case of product type, there were fewer store visits for e-cigarettes and nicotine pouches compared to cigars and cigarettes. Second, our generalizability was limited given that purchases were only attempted in three states, and the implementation of T21 will likely vary by state based on differences in state T21 laws such as tobacco product definitions, penalty structures, the number of compliance checks or inspections, and funding [28,29]. Third, our sample did not include vape shops in the first year of data collection in NJ. However, the study also has several strengths, including repeated measures over time across multiple sites and underage buyers.

As mentioned, only a few studies have focused on assessing retailer compliance with Tobacco 21 across various settings [13,14,15]. We believe that the processes studied here will be transferable to other contexts. Our team anticipates the publication and dissemination of results and different tools concerning the methods for evaluating the implementation of T21 and correlates of T21 compliance across several sites. The protocol developed will provide a method by which to demonstrate the impact of tobacco age of sale laws and potentially improve their implementation and enforcement. In the long term, the results of the evaluation of Tobacco 21 in three states will allow us to identify the factors that influence Tobacco 21 and develop strategies to improve compliance with the law and ultimately reduce youth access to tobacco.

## Figures and Tables

**Table 1:** Sample Characteristics by Site and Wave, 2019–2022.

Location and Data Collection		Sample	Total Buy Attempts
New Jersey			
	Wave 1, 08/19–03/20	86 tobacco retailers	780
	Wave 2, 06/21–10/21	60 tobacco retailers + 10 Vape shops	843
	Wave 3, 06/22–09/22	56 tobacco retailers + 10 Vape shops	1039
New York City			
	Wave 1, 10/19–03/20	92 tobacco retailers	390
	Wave 2, 10/21–07/22	50 tobacco retailers + 6 Vape shops	692
North Carolina			
	Wave 1, 01/22–08/22	51 tobacco retailers + 5 Vape shops	719
	Wave 2, 07/22–12/22	49 tobacco retailers + 4 Vape shops	663

**Table 2:** Buyer Characteristics by Site and Wave, 2019–2022.

New Jersey		
	W1	1 WF (20), 2 NWF (19, 19), 1 WM (20), 1 NWM (19)
	W2	2 WF (18, 20), 2 NWF (19, 20), 2 WM (19, 20), 2 NWM (18, 20)
	W3	3 WF (19, 19, 20), 3 NWF (18, 19, 19), 3 WM (18, 19, 20), 3 NWM (18, 19, 19)
New York City		
	W1	1 WF (20), 1 NWF (19), 1 WM (19), 1 NWM (19)
	W2	1 WF (20), 2 NWF (20, 18), 1 WM (20), 1 NWM (19)
North Carolina		
	W1	2 WF (20, 19), 5 NWF (20, 20, 20, 20, 19), 3 WM (20, 20, 20), 2 NWM (20, 19)
	W2	2 WF (19, 18), 2 NWF (19, 19), 2 WM (20, 19), 2 NWM (20, 19)

Specific time periods for Waves 1–3 can be found in Table 1. W = White; NW = Non-White; F = Female; M = Male; Buyers’ age in parentheses. Non-White included respondents who identified as Black, Hispanic, Middle Eastern, or Asian.

## Data Availability

Not applicable.
